# Editorial: Molecular imaging of cardiovascular diseases: current and emerging approaches in nuclear medicine

**DOI:** 10.3389/fnume.2023.1362018

**Published:** 2024-01-11

**Authors:** Jonathan Vigne, Giorgio Treglia

**Affiliations:** ^1^ Department of Nuclear Medicine, CHU de Caen Normandie, Normandy University, UNICAEN, Caen, France; ^2^ Department of Pharmacy, CHU de Caen Normandie, Normandy University, UNICAEN, Caen, France; ^3^ Normandie Université, UNICAEN, INSERM U1237, PhIND, Institut Blood and Brain @ Caen Normandie, Centre Cyceron, Caen, France; ^4^ Division of Nuclear Medicine, Imaging Institute of Southern Switzerland, Ente Ospedaliero Cantonale, Bellinzona, Switzerland; ^5^ Faculty of Biomedical Sciences, Università Della Svizzera Italiana, Lugano, Switzerland; ^6^ Faculty of Biology and Medicine, University of Lausanne, Lausanne, Switzerland; ^7^ Department of Nuclear Medicine and Molecular Imaging, Lausanne University Hospital (CHUV), Lausanne, Switzerland

**Keywords:** nuclear medicine, molecular imaging, nuclear cardiology, cardiovascular diseases, deep learning, myocardial perfusion

**Editorial on the Research Topic** Molecular imaging of cardiovascular diseases: current and emerging approaches in nuclear medicine

Cardiovascular diseases (CVDs), principally ischemic heart disease and stroke are the leading cause of death globally and represent a major cause of disability (1). Nuclear medicine approaches enable both the functional (2) and molecular imaging (3) of CVDs to confirm early diagnosis and guide the patient's management. Single photon emission computed tomography (SPECT) and positron emission tomography (PET) modalities rely on dedicated cameras and tracers that are used to detect various pathophysiological processes such as myocardial ischemia, myocardial viability, impaired myocardial innervation, vascular inflammation and myocardial fibrosis. The present Research Topic depicts cardiovascular applications of SPECT, PET and other medical imaging modalities to stratify the risk of cardiovascular events and improve the patients’ outcome.

In this Research Topic, Sun et al. investigated the impairment of blood flow to the tissues in the context of peripheral arterial disease (PAD) through the development of a PET tracer derived from Evans Blue (EB), named ^18^F-NEB, allowing *in vivo* labeling of albumin. They used an experimental model of hindlimb ischemic murine model to assess the potential of ^18^F-NEB to non-invasively monitor the blood perfusion in the early phase post-injection (p.i.) as well as the vascular permeability in the late phase p.i. corresponding to leakage of albumin from the vessel lumen. Interestingly, both blood perfusion and vascular permeability were measured over 14 days and compared with Laser Doppler and immunohistological results respectively. Moreover, Sun et al. evaluated whether early and late ^18^F-NEB PET imaging are capable of assessing blood perfusion and vascular permeability following treatment with vaascular endothelial growth factor (VEGF) known to induce angiogenesis and form more leaky capillaries. The potential of ^18^F-NEB PET appears promising for dual functional nuclear imaging and assessing therapeutic follow-up using VEGF.

Myocardial perfusion imaging (MPI) is the main clinical application in nuclear cardiology and mainly rely on measuring dedicated radiopharmaceuticals uptake in the left ventricle (LV). Hamzaraj et al. reported in this Research Topic a case of right ventricle overload due to primary pulmonary hypertension causing a D-shaped LV also called Movahed's sign on myocardial SPECT imaging that can alter interpretation of LV perfusion deficits.

In a study conducted at the Third Affiliated Hospital of Soochow University in Changzhou, China, published in this Research Topic, authors have identified epicardial fat volume (EFV) as a significant and independent risk factor for major adverse cardiovascular events (MACE) in individuals with suspected or known coronary artery disease (CAD) and normal left ventricular ejection fraction (LVEF). The study, led by Yang et al. shed light on the incremental prognostic value of EFV when combined with MPI in this particular population. The research, involving 290 Chinese inpatients, demonstrated that individuals with elevated EFV, defined as greater than 108.3 cm³, faced a 3.3-fold higher risk of experiencing MACE. Interestingly, even among those with normal MPI results, high EFV was associated with a significantly reduced event-free survival rate. The study suggests that incorporating EFV measurements into routine assessments, particularly in populations with normal LVEF, enhances the ability to predict cardiovascular events beyond usual risk factors and MPI. These findings underscore the importance of considering EFV as a novel and independent biomarker, offering a more comprehensive understanding of cardiovascular risk in Chinese populations with suspected or known CAD and normal LVEF. Future research may explore targeted interventions aimed at reducing EFV and improving outcomes in this high-risk group.

CAD remains a global health concern, necessitating multimodal innovative approaches for early detection and prevention. Herein, Lee et al. introduces an original model employing deep learning (DL) to predict significant coronary artery stenosis in asymptomatic individuals undergoing routine health check-ups. The research, based on a retrospective review of 11,180 cases, reveals that a neural network with multi-task learning outperformed traditional risk assessment tools. With an area under the curve (AUC) of 0.782, this DL-based model demonstrated a diagnostic accuracy of 71.6%, surpassing established methods such as the Pooled Cohort Equation, CAD consortium and updated Diamond-Forrester scores. Notably, authors identified personal education and monthly income levels as crucial features in the prediction model. This expansion beyond conventional risk factors showcases the model's adaptability to diverse data, reinforcing its potential for widespread applicability. Moreover, the study leveraged explainability tools like SHapley Additive ExPlanations (SHAP) to unravel the decision-making process of the DL model. This transparency in the model's predictions enhances its utility in clinical practice, providing valuable insights into the factors influencing the likelihood of obstructive CHD.

The conjunction between advanced technologies like DL and comprehensive datasets holds promise in the prevention of cardiovascular diseases. Nuclear medicine together with other medical imaging modalities will take part of cutting edge approaches to support clinical expertise for a more precise and effective screening tool.
